# Elevated blood pressure and analgesic overuse in chronic daily headache: an outpatient clinic-based study from China

**DOI:** 10.1186/1129-2377-14-51

**Published:** 2013-06-17

**Authors:** Qingqing Huang, Wangwen Li, Nan Li, Jing Wang, Ge Tan, Lixue Chen, Guangcheng Qin, Xiping Liang, Jiying Zhou

**Affiliations:** 1 Department of Neurology, The First Affiliated Hospital of Chongqing Medical University, Chongqing, China; 2 Laboratory Research Center, The First Affiliated Hospital of Chongqing Medical University, Chongqing, China; 3 Address:Medical College, Road No.1, Yuzhong District, Chongqing, China

**Keywords:** Acetaminophen, Analgesic overuse, Chronic daily headache, Elevated blood pressure

## Abstract

**Background:**

Many studies have reported that hypertension is common in chronic daily headache (CDH) and its subtype chronic migraine (CM), but the reason is still poorly understood. Our clinical literature review suggested that analgesic overuse may be associated with elevated blood pressure (BP), so we performed the present study to investigate the frequency of elevated BP and its link with analgesic overuse in CDH and its subtypes.

**Methods:**

A cross-sectional study was conducted in neurology outpatients with a diagnosis of CDH according to International Headache Society criteria. CDH patients were classified into CM and non-CM groups, and subclassified with or without analgesic overuse.

**Results:**

Elevated BP was present in 27.96% of CDH patients. Compared with non-CM patients, patients with CM had a longer duration of headache and more severe pain intensity, and a family history of headache and analgesic overuse were also more common, but the elevated BP frequency was not different between the two groups. Almost one-third of the patients had analgesic overuse; 96.8% of which comprised acetaminophen-containing agents. Those with analgesic overuse had a higher frequency of headache than those without analgesic overuse in both the CM and non-CM groups.

**Conclusions:**

Although the CM patients had a longer duration of headache, more severe intensity, the frequency of elevated BP wasn’t higher than non-CM group. Analgesic overuses maybe the reason of higher frequency of elevated BP in CDH and its subtypes. This may have predictive value for clinicians to improve CDH management.

## Background

CDH has a worldwide prevalence of 3% in the general population and is one of the most frequent consultation syndromes at headache clinics [1,2]. CDH lays a heavy burden on individuals and society, because of its frequency, severity and reduced quality of life. There is a relationship between hypertension and CDH and its subtype CM [3-5], which suggests that prevalence of hypertension is significantly higher in CDH or CM than in other headache groups.

CM is the most common subtype of CDH [6-9], Mathew et al. reported that patients with CDH transformed from originally episodic migraine had a higher possibility of hypertension [10], but the reason was poorly understood. Analgesic overuse is frequently identified as the most important risk factor for CM [11]. In CDH subtypes, CM patients are more likely to have analgesic overuse, which is a significant predictor of poor outcome.

Acetaminophen-containing agents are common in Chinese patients with analgesic overuse. In our previous study, 91.1% of patients with analgesic overuse used combination analgesics, in which acetaminophen and aspirin were the major components; 8.9% used nonsteroidal anti-inflammatory drugs (NSAIDs); and none used triptans or ergot alkaloids [12]. Acetaminophen is an over-the-counter drug, and it is recommended as first-line therapy for the management of chronic pain because of its greater cardiovascular safety and its low cost. Our review of the clinical literature suggested that acetaminophen is linked with a higher incidence of hypertension [13,14]. Treatment with acetaminophen results in a significant increase in mean systolic ambulatory BP in patients with coronary artery disease [15]. Aspirin dose is not significantly associated with hypertension [16,17]. Low-dose aspirin (75–325 mg/day) use is associated with a significant reduction in the risk of cardiovascular events [18,19].

There is no consensus about whether there is any difference in the frequency of hypertension in patients with CDH with or without analgesic overuse. One study found that the prevalence of hypertension in CDH did not significantly between with and without analgesic overuse [3]. However, another study reported that both CM with or without analgesic overuse had a stronger association with hypertension compared with other types of chronic headache (e.g. chronic post-traumatic headache) [5].

We performed the present study to investigate the difference in frequency of elevated BP in CM compared with other types of chronic headache; we also aimed to establish whether long-term use of acute analgesics increased BP in CDH patients and its subtypes, to provide effective strategies for the management of CDH and its subtypes.

## Methods

### Patient selection and study design

This was a cross-sectional study conducted at the Neurological Outpatient Department of the First Affiliated Hospital of Chongqing Medical University, China. All patients at least 18 years who consulted neurologists with a chief complaint of headache were included if they fulfilled the following criteria: daily or near-daily headache lasting >4 hours if not treated; occurring >15 days/month; fulfilling the Proposed Headache Classification for Chronic Daily Headache described by Silberstein and Lipton [20-22].

The Silberstein and Lipton criteria divide CDH into four subtypes: chronic or transformed migraine; chronic tension-type headache; new daily persistent headache; and hemicrania continua. These are then subclassified as with or without analgesic overuse.

To be classified as CM, the patients should satisfy the following conditions: Headache on ≥15 days/month for at least 3 months, Occurring in a patient who has at least 8 days/month fulfilling criteria 1.1 (migraine without aura), not attributed to another causative disorder. Another three subtypes of CDH were classified as non-CM in order to investigate differences in frequency of elevated BP in CM compared with other chronic headache types.

The diagnosis of analgesic overuse adopted the criteria proposed by Silberstein and Lipton. Patients who met the following criteria for at least 3 months in the previous year were considered to have analgesic overuse: intake of simple analgesics ≥15 days/month or combination medications ≥10 days/month for at least 3 months.

A face-to-face interview method was used for data collection. Each participant was examined and given a detailed structured self-administered questionnaire by a neurologist [12,23-25]. The questionnaire included the following components: demographic characteristics, clinical features of headache (onset age, duration, frequency, intensity, location, accompanying symptoms, and aggravation after activity); history of head injury; past medical history and drug intake for headache; family history of headache and history of cerebrovascular comorbidity in first-degree relatives. All patients were asked the above questions according to the questionnaire.

Headache intensity was evaluated using a 0–10 visual analog scale (0: no pain; 1–3: mild; 4–6: moderate; 7–9: severe; 10: maximal pain). BP was measured according to a standardized method as in other cross-sectional studies [26,27]. The participants were seated, and BP was measured 3 times after at least 5 minutes rest, and the average value was recorded. Elevated BP was diagnosed in patients with systolic BP (SBP) ≥140 mmHg or diastolic BP (DBP) ≥90 mmHg, or use of antihypertensive agents.

Second headache was excluded using appropriate tests if necessary. Headache attributed to the use of acute medication for other conditions was also excluded. The neurologists were blinded to patient identity when collecting information about demographic characteristics, headache, and neurological examination.

The study protocol was approved by the Ethical Committee of the First Affiliated Hospital of Chongqing Medical University. Informed consent was given by the patients who agreed to participate in the study.

### Statistical analysis

Statistical analysis was performed using SPSS version 17.0. The characteristics of the study population were analyzed using descriptive statistics. Measurement data variables were expressed as mean ±standard deviation (SD). The χ^2^ and *t* tests were used for comparison when appropriate. The aim of collecting information of the onset age of headache, intensity, accompanying symptoms and whether the headache aggravated after activity was to diagnose, so these were not included in the analyses. *P* < 0.05 was considered statistically significant, and all statistical tests were two-sided.

## Results

During the study period (20 July 2011 to 30 December 2011), 10 315 consecutive patients visited the neurological outpatient of the First Affiliated Hospital of Chongqing Medical University. Among the 1327 patients (12.9%) who cited headache as their chief complaint, 1219 were able to participate in the study (response rate 91.9%). Among the 311 patients who suffered from CDH, 6 were excluded because of incomplete information, and 1 for overuse of addictive drugs. Finally, a total of 304 CDH patients were recruited: 109 with CM (53 with and 56 without analgesic overuse), and 195 non-CM (42 with and 153 without analgesic overuse).

Table 1 summarizes the demographics of the study population. Women accounts for 75% in CDH patients, more common in CM group than in non-CM group (84.2% in CM vs69.4% in non-CM, p=0.004). Compared with non-CM group, CM patients had a longer duration of headache than non-CM patients (*P* < 0.000); pain intensity was more severe,more patients had a family history of headache; and more were more likely to have analgesic overuse,although the average number of headache days per month was higher in non-CM than in CM patients.

**Table 1:** The demographics of the study population

**Variable**	**CM (n=109)**	**non-CM (n=195)**	**P value**
Age (Y) (mean±SD)	46.64±12.13	47,58±14.03	0.553
Female (n, %)	92(84.2)	136(69.4)	0.004
Female to male ratio	5.33	2.27	
BMI (mean±SD)	22.69±3.91	22.15±3.53	0.231
Education level (n, %)			0.531
Primary school or Less	87(76.3)	139(70.9)	
High school or technical school	18(15.8)	35(17.9)	
University	9(7.9)	22(11.2)	
Elevated Blood Pressure (n, %)	32(29.4)	53(27.2)	0.685
DBP	74.98±18.15	78.02±11.28	0.114
BP	116.09±28.19	119.17±18.51	0.251
Smoking (n, %)	13(11.4)	31(15.8)	0.283
Alcohol consumption (n, %)	3(2.6)	9(4.6)	0546
Duration of headache history (y, mean±SD)	13.41±11.88	7.35±9.20	0.000
pain intensity (mean±SD)	6.50±1.51	4.91±1.70	0.000
Average headache days per month (day, mean±SD)	23.18±7.88	25.77±6.08	0.001
Average days per month had analgesic overuse	11.26±11.76	6.29±10.74	0.000
Analgesic overuse (n, %)	53(48.6)	42 (21.5)	0.000
Family headache history (n, %)	58(51.3)	54(27.8)	0.000
Cerebrovascular disease history (first degree relative) (n, %)	14(12.4)	14(7.2)	0.129

Alcohol consumption and smoking indicated daily or nearly-daily drinking or smoking;Family headache history and Cerebrovascular disease history indicated the first degree relative headache and cerebrovascular disease history; cerebrovascular disease included stroke (cerebral hemorrhage, infarction) and transient ischemic attack (TIA) et al. measurement data variables were expressed as mean±SD; CDH—chronic daily headache; SD—standard deviation;BMI—Body Mass Index. P values were two tailed and p value <0.05 was defined as statistical significance.

With regard to the elevated BP frequency, BMI, the frequency of low education level, family cerebrovascular disease history (first degree relative), and smoking and alcohol consumption, there were no significant differences between the two groups (P > 0.05).

Many modifiable factors influenced CDH progression and development of CM, analgesic overuse was frequently identified as the most important factor, and was seen in 95 (31.3%) of CDH patients (Table 2). We analyzed the effect of analgesic overuse on CM and other CDH types (as showed in Table 3). In the CM group, there was no significant difference in age, sex, BMI, frequency of low education level, and smoking between patients with or without analgesic overuse (*P* > 0.05). However, those with analgesic overuse had a longer duration of headache and more severe pain intensity than those without analgesic overuse. The frequency of elevated BP was also higher in patients with analgesic overuse than in those without analgesic overuse (*P* = 0.011). Among 85 patients with CDH and elevated BP, 19 were taking antihypertensive agents, and the mean BP was 126.63/81.05 mmHg. Although both DBP and SBP were higher in those with analgesic overuse, only SBP was significantly higher (*P* = 0.04).

**Table 2:** The occurrence of analgesic overuse in CM and non-CM patients

	**CM (n, %)**	**Non-CM (n, %)**	**Total (n, %)**	**P value**
Analgesic overuse				
Yes	53 (48.6)	42 (21.5)	95 (31.25)	0.000*
No	56 (51.4)	153 (78.5)	209 (68.75)	
Total	109	195	304	

*p value -the occurrence of analgesic overuse in CM group compared with in non-CM, p<0.05 had a statistic difference.

**Table 3:** The demographics of the subgroups of two groups

**Variable**	**CM (n=109)**			**non-CM (n=196)**		
Analgesic overuse	without	With	p	without	With	P
Age (Y) (mean±SD)	44.66±15.36	48.76±9.92	0.096	46.41±4.88	52.12±9.66	0.019
Female (n, %)	47(79.7)	49(89.1)	0.204	104(68.0)	31(73.8)	0.572
Female to male ratio	3.92	8.17		2.12	2.82	
BMI(mean±SD)	22.71±4.53	22.67±3.17	0.957	22.02±3.58	22.54±3.31	0.379
Education level (n,%)			0.227			0.017
Primary school or Less	42(71.2)	45(81.8)		102(66.7)	36(85.7)	0.378
High school or technical school	10 (16.9)	8(14.5)		29(19.0)	6(14.3)	
University or above	7(11.9)	2(3.6)		22(14.4)	0	
EBP frequency (n,%)	10(17.9)	22(41.5)	0.011	36(23.5)	17(40.5)	0.033
DBP	73.23±14.62	78.53±18.13	0.092	77.33±10.86	80.5±12.53	0.141
SBP	111.75±22.98	122.02±28.87	0.040	116.87±16.88	127.57±21.75	0.005
Smoking (n, %)	8(13.6)	5(9.1)	0.561	25(16.3)	6(14.3)	1
Alcohol consumption (n, %)	2(3.4)	1(1.8)	0.527	6(3.9)	2(4.8)	0.545
Duration of headache history (y, mean±SD)	9.57±10.71	17.53±11.78	0.000	6.71±9.37	9.62±8.37	0.057
headache frequency/month in the recent 3 months (day, mean±SD)	23.25±8.36	23.09±7.42	0.912	25.73±6.07	25.93±6.21	0.858
Headache pain intensity (mean±SD)	6.21±1.37	6.81±1.60	0.034	4.88±1.68	5.05±1.79	0.591
Family headache history (n, %)	28(47.5)	30(55.6)	0.453	36(23.5)	18(43.9)	0.017
Cerebrovascular disease history (first degree relative) (n,%)	10(16.9)	4(7.4)	0.158	11(7.2)	3(7.3)	0.601

Analgesic overuse indicated compound analgesic >10 days per month or simple analgesic >15 days per month, lasted for at least 3 months in recently. Family headache history and Cerebrovascular disease history indicated the first degree relative headache and cerebrovascular disease history; cerebrovascular disease included stroke (cerebral hemorrhage, infarction) and transient ischemic attack (TIA) et al. P values were two tailed and statistical significance was defined as p value <0.05. CDH—chronic daily headache; RR(95%CI)—relative risk(95% confidence interval) ;SD—standard deviation.BMI—Body Mass Index.

In the non-CM group, patients with analgesic overuse were significantly older than those without analgesic overuse (*P* = 0.019). Except for age, they were similar in sex, BMI, frequency of low education level, smoking, headache character, and history of cerebrovascular disease in first-degree relatives (*P* > 0.05). Similar to CM patients, non-CM with analgesic overuse had a higher frequency of elevated BP compared with those without analgesic overuse (*P* = 0.033).

In the 95 CDH patients with analgesic overuse, 92 were taking compound analgesics or had compound and simple analgesics together; 3 used one simple analgesic (ibuprofen or naproxen), in total, 7 patients had NSAIDs(ibuprofen or naproxen). The most frequently taken agents included “anti-headache flour”, coldrex, acetaminophen and “pain relieved pill”. Except for ibuprofen and naproxen (NSAIDs), all contained acetaminophen or both aspirin and acetaminophen contained.

Table 4 showed the odds ratio and P value of the risk of elevated BP frequency in CDH and its subtypes with analgesic overuse compared with those without analgesic overuse. For all CDH patients, those with analgesic overuse had a higher frequency of elevated BP than those without analgesic overuse (*P* = 0.001). The risk of elevated BP in CDH with analgesic overuse was 2.468 times higher than in those without analgesic overuse (OR = 2.468, 95% CI = 1.46–4.17). In CM patients with analgesic overuse, the risk of elevated BP was 3.265 times higher than in those without analgesic overuse (OR = 3.265, 95% CI = 1.36–7.83). In the non-CM group, the OR was 2.21 (95% CI = 1.075–4.543).

**Table 4:** The odds ratio of elevated BP frequency in CDH patients with analgesic overuse compared with those without analgesic overuse

**Variable**		**Elevated BP frequency**		
	**Total(n)**	**N (%)**	**P Value**	**OR (95% CI)**
Compared with those without analgesic overuse				
All patients with analgesic overuse	93	39 (41.1)	0.001	2.468 (1.46-4.17)^1^
CM with analgesic overuse	53	22 (41.5)	0.011	3.265 (1.36-7.83)^2^
Non-CM with analgesic overuse	42	17 (40.5)	0.033	2.21 (1.075-4.543)^3^

^1^ was all CDH patients with analgesic overuse compared with those without, ^2^ indicated in CM group those with analgesic overuse compared with those without, ^3^ indicated in non-CM group those with analgesic overuse compared with those without.

## Discussion

The relationship between headache and hypertension is still controversial [5,11,27-31]. Some studies in patients with mild hypertension reported that there was no association between the occurrence of headache and BP variation [28], and most individuals with headache had BP similar to those who did not [29]. However, other studies had the opposite view. Cooper et al. suggested that headache was common in mild to moderate hypertension and could be reduced by treatment [30]. Angiotensin-converting enzyme (ACE) inhibitor use was associated with a significantly lower risk of headache in patients with hypertension [31]. Ziegler et al. found that hypertension had an association with severe headache in women [32]. Additionally, in major tertiary care centers, prevalence of hypertension in CDH patients was 16.2%, which was significantly higher than in those with episodic migraine and TTH [3]. High BP(CM 33.7% vs. EM 27.9%; OR (95% CI)=1.2 (1.03 to 1.47), p=0.02)) occurred with greater frequency in CM than in EM[2], in spite of CM with or without analgesic overuse (OR=2.9/6.9 compared with EM respectively) [5].

In our study, we also found that CDH had a strong association with elevated BP (OR (95% CI) = 2.468(1.46–4.17)). The elevated BP frequency in CDH patients was 27.96%, while the prevalence of hypertension in adults reached 18.8% in Mainland China in 2002 [33]. Pietrini et al. evaluated 1486 patients with headache and found that the prevalence of hypertension was 28%, which was significantly higher than in all age groups of the general population [26]. The frequency was similar with our study.

CM is the most common subtype of CDH [6-9], as in our study. However, in a study in Taipei, CTTH was the most common subtype of CDH in older patients [11], the study included patients aged >65 years, with a mean age of 73.7 ± 6.9 years, which is older than in the present and other previous studies. Mathew et al. reported that CDH patients who transformed from episodic migraine had a higher likelihood of hypertension [10]. Bigal et al. showed that prevalence of hypertension in CM patients was 6.9 times higher than in episodic migraine patients, and 5.1 times higher than in those with chronic post-traumatic headache [5].

While compared with non-CM, the frequency of elevated BP didn’t increased in CM, there was no significantly difference between two groups, although the CM patient had a longer duration of headache, a more severe intensity. Maybe there didn’t exist a causal relationship between the elevated BP frequency and duration of headache, intensity.

In many studies, analgesic overuse has been identified as the most important risk factor for CDH and development of CM [3,11]. In our study, the occurrence of analgesic overuse in all CDH patients was 31.3%, 48.6% in CM patients and 21.5% in non-CM patients; the occurrence of analgesic overuse in CM was significantly higher than in non-CM patients. It seems that patients having a longer duration of headache, more severe pain intensity were more likely to overused analgesic than patients having a frequency of headache days /month.

Patients with analgesic overuse had a higher frequency of elevated BP. Elevated BP was presented in 61.1% of CDH patients with analgesic overuse. Both In CM and non-CM groups, the frequency of elevated BP in patients with analgesic overuse were significantly higher than in those without analgesic overuse (p<0.05). It seems that the frequency of elevated BP is associated with analgesic overuse; maybe there exists a causal relationship between elevated BP and analgesic overuse.

Acetaminophen and aspirin were the most common components in our patients with analgesic overuse. NSAIDs were taken by 8.9% of patients in our previous study [12], and 7.4% patients had coadministration of NSAIDs with other analgesics in the present study. Some studies reported that acetaminophen was linked with a higher incidence of hypertension [13,14]. Treatment with acetaminophen resulted in a significant increase in mean ambulatory SBP in patients with coronary artery disease [15]. Epidemiological studies such as that by Forman et al. demonstrated that men who took acetaminophen 6 or 7 d/week had an increased relative risk for hypertension compared with those taking NSAIDs [34]. Additionally, in the Nurses’ Health Study I and II, the multivariable-adjusted relative risk of hypertension in women who took >500 mg/day acetaminophen was increased almost 2-fold compared with women who did not use acetaminophen [16]. Aspirin dose was not significantly associated with hypertension [16,35]. Low dose aspirin (75–325 mg/day) use is associated with a significant reduction in the risk of cardiovascular events [18,19]. In a meta-analysis of NSAIDs and acetaminophen, aspirin use was investigated, although slight increases in BP were noted, the CIs were wide and not statistically significant [17].

Other epidemiological and small interventional studies have shown that NSAIDs were associated with an increased risk of hypertension [35,36]. In a recent analysis of women who provided more extensive data on analgesic usage, a significant, dose-dependent increase in risk of hypertension was observed among those using NSAIDs irrespective of the reason [16]. In the Physicians’ Health Study, men who estimated taking ≥61 NSAID tablets per year had a RR for hypertension of 1.05 compared with those who took none.

Although both acetaminophen and NSAIDs were reported elevating BP. In our patients with analgesic overuse, compound analgesics were the most commonly used: 96.8% of those who overused analgesics took “anti-headache flour” or coldrex. The main components of “Anti-headache flour” were aspirin and acetaminophen. The main component of coldrex and other compound analgesic was acetaminophen. Simple analgesics, such as ibuprofen and naproxen, were usually taken by patients upon the advice of a doctor, instead of “anti-headache flour” or coldrex after they had had analgesic overuse. Few patients took opioids or amitriptyline to alleviate headache. Almost no patients used ergotamine and triptans.NSAIDs only accounts for 7.4%. This is similar to a previous study in Mainland China [12].

In a study in Taipei, 7.5% of patients with analgesic overuse took ergotamine and caffeine, and 24% to liquid common cold medications, but overuse of triptans was rare [37]. In Japan, combination analgesics were a common choice in patients with analgesic overuse; few patients took ergotamine and triptans and none overused opioids [38]. However, in Germany, both ergot alkaloids and triptans were frequently overused analgesics [39].

Our results suggest that analgesic overuse is the main reason for patients with CDH having a higher frequency of elevated BP, and acetaminophen might play an important role. Pietrini et al. also found that patients with analgesic overuse had a high prevalence of hypertension in their headache center (60.6%) [24]. In 2 large, prospective, female cohorts [35,36], an association between the frequency of analgesic use and risk of developing hypertension was reported. However, Gipponi et al. reported that the prevalence of hypertension did not differ significantly between CDH patients with or without analgesic overuse [3]. This needs further study.

Low education level plays an important role in CM with analgesic overuse. In one large cohort of female health professionals, low socioeconomic status was associated with an increased frequency of migraine attack [40]. The proportions of patients using prophylactic medication and having consulted a neurologist were smaller among those who only had elementary school education compared with higher education; Those with a lower level of education also had a higher number of days per month with headache and with analgesic overuse than those with higher educational level [41]. A multivariate analysis showed that socioeconomic factors such as having a low level of education and/or a low household income were associated with analgesic overuse [42]. Migraine patients with low socioeconomic status may have a risk of developing medication overuse headache, The duration of education was shorter in migraine patients with low income [43]. Low education level plays an important role in analgesic overuse, so we need more focus on patients with low education level in future.

Patients with comorbidity of hypertension and migraine have a higher prevalence of cerebrovascular events [27]. Hypertension amplifies the effect of migraine on the vascular wall, which further enhances cerebrovascular endothelial dysfunction [44]. Patients with hypertension using ACE inhibitors have a significantly lower risk of headache [32]. Antihypertensive treatment can also decrease the incidence of headache [15]. All the above results emphasize the importance of the control of hypertension in management of CDH.

The study had some limits. First, this was a cross-sectional study, BP was evaluated at only one visit; therefore, we did not diagnose hypertension, and only defined the condition of elevated BP. Second, the findings study was conducted in hospital outpatient; therefore, the results should not be extrapolated to the general population.

## Conclusions

The present study found that CDH patients with analgesic overuse had a higher frequency of elevated BP than those without analgesic overuse. Acetaminophen-containing analgesics were the most common agents in present study, which maybe the reason of elevated BP in CDH and its subtype. These maybes provide basis theory for the hypertension management in CDH, especially in CM.

## Abbreviations

CDH: Chronic daily headache; CM: Chronic migraine; BP: Blood pressure; BMI: Body mass index; CI: Confidence interval; SD: Standard deviation.

## Competing interests

On behalf of all authors, the corresponding author states that they have no competing interests.

## Authors' contributions

QH carried out all process of the manuscript. WL, NL, JW and XL participated in the collection of data in the neurology outpatient. GT and LC drafted the manuscript, instructed the statistical analysis. JZ participated in the design of the study and coordination. GQ participated in its revision and helped to draft the revised manuscript. All authors read and approved the final manuscript.
